# Impact of Tumor Side on Clinical Outcomes in Stage II and III Colon Cancer With Known Microsatellite Instability Status

**DOI:** 10.3389/fonc.2021.592351

**Published:** 2021-03-30

**Authors:** Mehmet Akce, Katerina Zakka, Renjian Jiang, Shayla Williamson, Olatunji B. Alese, Walid L. Shaib, Christina Wu, Madhusmita Behera, Bassel F. El-Rayes

**Affiliations:** ^1^ Department of Hematology and Medical Oncology, Winship Cancer Institute, Emory University, Atlanta, GA, United States; ^2^ Department of Internal Medicine, Wellstar Health System, Atlanta Medical Center, Atlanta, GA, United States; ^3^ Winship Research Informatics, Winship Cancer Institute, Emory University, Atlanta, GA, United States

**Keywords:** colon cancer, microsatellite instability, tumor side, stage II colon cancer, stage III colon cancer

## Abstract

**Background:**

Tumor sidedness as a prognostic factor in advanced stage colon cancer (CC) is well established. The impact of tumor sidedness on the clinical outcomes of stage II and III CC has not been well studied.

**Methods:**

The National Cancer Database (NCDB) was utilized to identify patients with pathological stage II and III primary adenocarcinoma of the colon from 2010 to 2015 using ICD-O-3 morphology and topography codes: 8140-47, 8210-11, 8220-21, 8260-63, 8480-81, 8490 and C18.0, 18.2,18.3, 18.5,18.6, 18.7. Univariate (UVA) and multivariable (MVA) survival analyses and Kaplan–Meier Curves with Log-rank test were utilized to compare overall survival (OS) based on tumor location and treatment received.

**Results:**

A total of 35,071 patients with stage II (n = 17,629) and III (n = 17,442) CC were identified. 51.3% female; 81.5% Caucasian; median age 66 (range, 18–90). Majority of stage II and III tumors were right sided, 61.2% (n = 10,794) and 56.0% (n = 9,763). Microsatellite instability high (MSI-H) was more common in stage II compared to III, 23.3% (n = 4,115) *vs* 18.2% (n = 3,171) (p < 0.0001). In stage II MSI-H CC right was more common than left, 78.3% (n = 3223) *vs* 21.7% (n = 892). There was no significant difference in survival between stage II MSI-H left *vs* right (5-year OS 76.2 *vs* 74.7%, p = 0.1578). Stage II MSS CC right was more common than left, 56.0% (n = 7571) *vs* 44.0% (n = 5943), and survival was better in the left *vs* right (5-year OS 73.2 *vs* 70.8%, p = 0.0029). Stage III MSI-H CC was more common in the right than in the left, 75.6% (n = 2,397) vs 24.4% (n = 774) and survival was better in the left (5-year OS 62.5 *vs* 56.5%, p = 0.0026). Stage III MSS CC was more common in the right than in the left, 51.6% (n = 7,366) *vs* 48.4% (n = 6,905), and survival was better in the left *vs* right (5-year OS 67.0 *vs* 54.4%, p < 0.001).

**Conclusion:**

Survival was better in left sided tumors compared to right in stage II MSS, stage III MSS, and stage III MSI-H CC.

## Highlights

Given the paramount importance of MSI status in locoregional colon cancer (CC) management and the propensity for MSI-H tumors for the right side, it is imperative to analyze the impact of tumor sidedness with known MSI status. This large national cancer database analysis revealed that survival was better in left sided tumors compared to right in stage II MSS, stage III MSS and stage III MSI-H CC. Survival benefit from adjuvant chemotherapy was observed in all patients except stage II left sided MSI-H CC patients.

## Introduction

Colorectal cancer is the third most common cancer and third leading cause of cancer related mortality in the United States (US) (1). It is estimated that 104,610 new cases of colon cancer (CC) will be diagnosed in the US in 2020. Two thirds of patients present with locoregional disease, and primary tumor location could have a significant impact on the prognosis in CC across all stages (1–3). The predictive role of tumor sidedness was described in the locoregional (4–6) and metastatic setting (7, 8). Embryologic and physiologic differences exist between the left and right sides of the colon. The portion of the large intestine from the cecum to the proximal two thirds of the transverse colon is derived from the midgut, and the distal third of the transverse colon to the upper anal canal is derived from the hindgut (9, 10). Clinicopathological characteristics of left- and right-sided colon tumors differ significantly (2, 3, 9, 11). Right-sided CCs are more likely to be diploid, exophytic, microsatellite instability-high (MSI-H), have mucinous histology and CpG island methylation; on the other hand, left-sided CCs are more often aneuploidy, infiltrating lesions, present with symptoms of obstruction and have chromosomal instability (5, 10, 12–14). Significant differences exist in gene expressions between tumors of the right and left side of the colon (15–17). Right-sided tumors are characterized by defective MMR genes, mutations of KRAS and BRAF, and microRNA-31, whereas left-sided CC is associated with CIN, p53, APC, NRAS, ERBB2 microRNA-146a, microRNA-147b, and microRNA-1288 (5, 18).

Microsatellite instability (MSI) is an independent predictor of overall survival (OS) and MSI-H tumors have a better overall prognosis (19–23) and significantly decreased risk of metastasis (22) compared to microsatellite stable (MSS) tumors of the colon. It is estimated that 20–25% of right-sided stage II CCs are MSI-H; MSI-H tumors of the left colon are far less common, across all stages (17, 20, 24–27). The prognostic role of tumor-sidedness has been extensively studied in locoregional CC; however, MSI status was not included in these studies (4–6). Given the paramount importance of MSI status in locoregional CC management and the propensity for MSI-H tumors for the right side, it is imperative to analyze the impact of tumor sidedness with known MSI status. The aim of this study is to evaluate the impact of primary tumor side, left-sided (L) *versus* right-sided (R), on clinical outcomes based on known MSI status in patients with stage II and III CCs. We also sought to determine whether tumor side based on known MSI status is predictive of adjuvant chemotherapy (AC) benefit in stage II and III CCs.

## Patients and Methods

**Figure 1:**
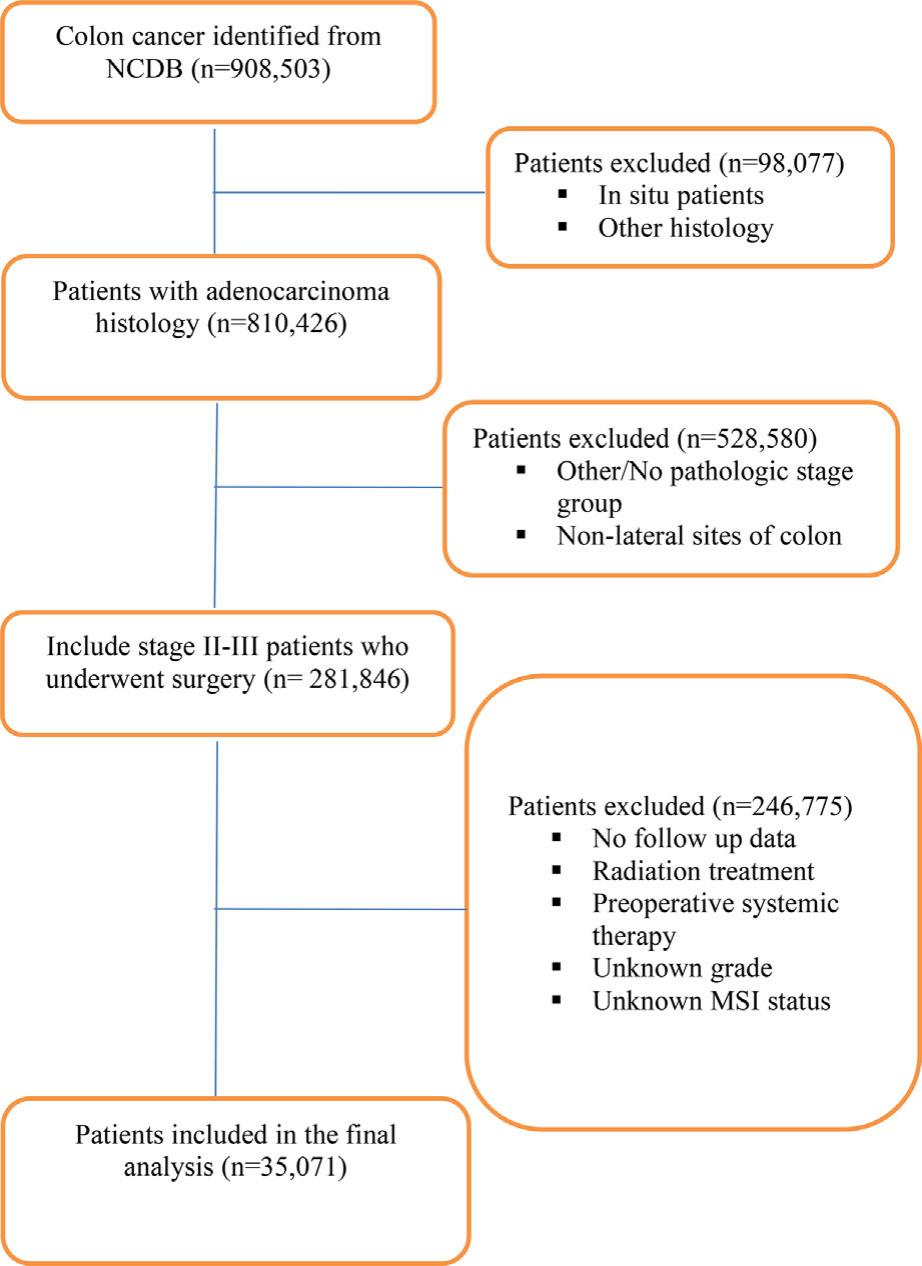
Consort diagram.

The National Cancer Database (NCDB) was utilized to identify patients with pathological stage II and III primary adenocarcinoma of the colon between years 2010 and 2015 who underwent resection. The NCDB contains clinical and demographic information on 70% of all incident cancers in the United States from >1,500 Commission-on-Cancer-accredited cancer centers. It is a joint quality improvement initiative of the American College of Surgeons Commission on Cancer and the American Cancer Society. Eligibility was obtained using the following ICD-O-3 morphology and topography codes: 8140-47, 8210-11, 8220-21, 8260-63, 8480-81, 8490 and C18.0, 18.2, 18.3, 18.5, 18.6, 18.7 (**Figure 1**). Since portions of the transverse colon are within the left and right sides of the colon, tumors of the transverse colon were excluded. Patients that received neoadjuvant systemic/radiation therapy and adjuvant radiation were also excluded. Microsatellite stability status was divided into microsatellite stable (MSS) which included MSI stable (code 020) and MSI unstable low positive (code 040). Microsatellite unstable (MSI-H) status included MSI unstable high positive (code 050) and MSI unstable positive (code 060). Tumors without known MSI status were excluded. The primary outcome was OS difference between patients with right-sided tumors compared to left-sided tumors based on MSI status. The secondary outcome was OS of patients who received adjuvant chemotherapy compared to patients that received no treatment stratified by tumor side and MSI status. The following patient-specific covariates were included: sex, race, facility type, insurance status, year of diagnosis, AJCC pathologic stage, primary site, surgical margin status, microsatellite stability status, regional lymph nodes examined, Charlson–Deyo score, chemotherapy, type of surgery, age at diagnosis, and tumor size (**Table 1**). No ethical approval was required for the study as de-identified patient information in the NCDB is legally accessible to the public.

**Table 1:** Baseline clinicopathological characteristics.

Variable	Level	Entire cohort N (%) = 35,071
**Sex**	Male Female	1,7067 (48.7) 1,8004 (51.3)
**Race**	African American Other/Unknown Caucasian	4,372 (12.5) 2,118 (6.0) 28,581 (81.5)
**AJCC Pathologic Stage Group**	II III	17,629 (50.3) 17,442 (49.7)
**Primary Site**	Right Left	20,557 (58.6) 14,514 (41.4)
**Microsatellite Instability (MSI) Status**	MSS MSI-H	27,785 (79.2) 7,286 (20.8)
**Regional Lymph Nodes Examined**	>=12 <12 Unknown/missing	33,038(94.3) 1,947 (5.6) 86 (0.1)
**Pathological T stage**	T1	617 (1.8)
	T2	1,538 (4.5)
	T3	26,197 (74.7)
	T4	160 (0.5)
	T4A	4,429 (12.6)
	T4B	2,022 (5.8)
	TX	63 (0.1)
**Year of Diagnosis**	2010–2012 2013–2015	13,834 (39.4) 21,237 (60.6)
**Facility Type**	Community Cancer Program Comprehensive Community Cancer Program Academic/Research Program Integrated Network Cancer Program Other specified types of cancer programs	3,011 (8.6) 13,860 (39.5) 10,867 (31.0) 5,797 (16.5) 1,536 (4.4)
**Insurance Status**	Government Insurance Unknown Not Insured Private Insurance	19,712 (56.2) 360 (1.0) 1,314 (3.7) 13,685 (39.0)
**Charlson-Deyo Score**	0 1 2+	24,272 (69.2) 7,538 (21.5) 3261 (9.3)
**Tumor Size (cm)**	Mean Median Minimum Maximum Std Dev	5.32 4.90 0.00 98.90 3.73
**Chemotherapy**	No* Yes Unknown	17,774 (50.7) 16,476 (47.0) 821 (2.3)
**Surgery at Primary Site**	Partial colectomy Subtotal colectomy/hemicolectomy Surgery NOS Total colectomy	11,422 (32.6) 22,425 (63.9) 37 (0.1) 1,187 (3.4)
**Age at Diagnosis**	Mean Median Minimum Maximum Std Dev	65.05 66.00 18.00 90.00 14.35

*Includes 3,709 (0.1%) patients who were recommended but not administered chemotherapy.

### Statistical Analysis

The clinical and demographic characteristics of the patients were summarized using descriptive statistics as appropriate for variable type and distribution. For numeric covariates, the mean, median, range, and standard deviation were presented. Frequency and its percentage were generated for categorical variables. For descriptive statistics, chi-square tests were performed for categorical variables and ANOVA for continuous variables. OS was defined as months from diagnosis to death or last contact, where those who were alive were censored at last contact. OS was estimated using the Kaplan–Meier method, and patient variables were compared across OS using log-rank tests. Univariate Cox proportional hazards models were fit for OS as a function of primary site, chemotherapy, microsatellite status, sex, Charlson–Deyo score, race, year of diagnosis, tumor size, facility type, insurance status, and age at diagnosis. A multivariable Cox model was fit for OS as a function of the previously mentioned covariates. Model assumptions were assessed and verified. All analyses were done using SAS 9.4 (SAS Institute, Inc., Cary, North Carolina) with a significant level of 0.05.

## Results

### Patient Demographics and Tumor Characteristics

A total of 35,071 patients with resected pathological stage II (n = 17,629) and III (n = 17,442) CCs were identified (**Figure 1**). Baseline clinicopathological characteristics are summarized in **Table 1**. The median age at diagnosis was 66 (range, 18–90) years, with females accounting for 51.3%. About 81.5% were Caucasian; 48.1% of the patients were treated at community practices, and 31.0% were treated at academic or research cancer centers. Adjuvant chemotherapy was administered in 47.0% of patients. Insurance coverage was mostly government (56.2%) in comparison to private insurance (39.0%) and no insurance (3.7%). A higher number of patients were diagnosed between 2013 and 2015 (60.6%) compared to 2010 and 2012 (39.4%). Charlson–Deyo score was 0 for most patients (69.2%) compared to ≥1 in 30.8% of patients. Median tumor size was 4.90 cm (**Table 1**).

The majority of stage II and III tumors were R (II: 61.2%, n = 10,794 and III: 56.0%, n = 9,763). MSS accounted for 79.2% and MSI-H for 20.8%. MSI-H was more common in stage II compared to III (II: 23.3%, n = 4,115 *vs* III: 18.2%, n = 3,171) (p < 0.0001). MSI-H CC was more common on the right side in stage II (R: 78.3%, n = 3,223 *vs* L: 21.7%, n = 892) and stage III (R: 75.6%, n = 2,397 *vs* L: 24.4%, n = 774). Similarly, in MSS CC right-sided was more common than left in stage II (R: 56.0%, n = 7571 *vs* L: 44.0%, n = 5,943) and stage III (R: 51.6%, n = 7,366 *vs* L: 48.4%, n = 6,905) CC. Baseline clinicopathological characteristics stratified by tumor side are summarized in **Table 2**.

**Table 2:** Univariate association with tumor side.

	Covariate	Level	Primary Site		
			Right N = 3223	Left N = 892	P-value
Stage II MSI-H Patients	Sex	Male Female	1,268 (39.34) 1,955 (60.66)	486 (54.48) 406 (45.52)	<.001
	Race	African American Other Caucasian	247 (7.66) 124 (3.85) 2,852 (88.49)	85 (9.53) 60 (6.73) 747 (83.74)	<.001
	Insurance Status	Government Unknown Not insured Private	2,161 (67.05) 25 (0.78) 84 (2.61) 953 (29.57)	455 (51.01) 6 (0.67) 40 (4.48) 391 (43.83)	<.001
	Surgical Margin Status	No Yes Unknown	3,115 (96.65) 103 (3.2) 5 (0.16)	859 (96.3) 30 (3.36) 3 (0.34)	0.535
	Charlson–Deyo score	0 1 2+	2,059 (63.88) 773 (23.98) 391 (12.13)	649 (72.76) 175 (19.62) 68 (7.62)	<.001
	Chemotherapy	No Yes Unknown	2,745 (85.17) 404 (12.53) 74 (2.3)	667 (74.78) 198 (22.2) 27 (3.03)	<.001
	Type of Surgery	Partial colectomy Subtotal colectomy/hemicolectomy Surgery NOS Total Colectomy	389 (12.07) 2,748 (85.26) 1 (0.03) 85 (2.64)	457 (51.23) 390 (43.72) 2 (0.22) 43 (4.82)	<.001
	Age at Diagnosis	Mean Median Min Max Std Dev	69.28 71 18 90 14.22	62.02 63 19 90 15.66	<.001
	Tumor Size (cm)	Mean Median Min Max Std Dev	6.39 6 0.2 98.9 3.53	6.08 5.5 0.5 98.9 5.4	0.040
	**Covariate**	**Level**	**Right N = 7571**	**Left N = 5943**	**P-value**
Stage II MSS Patients	Sex	Male Female	3,722 (49.16) 3,849 (50.84)	3,131 (52.68) 2,812 (47.32)	<.001
	Race	African American Other Caucasian	1,006 (13.29) 377 (4.98) 6,188 (81.73)	696 (11.71) 439 (7.39) 4,808 (80.9)	<.001
	Insurance Status	Government Unknown Not insured Private	4,802 (63.43) 75 (0.99) 232 (3.06) 2,462 (32.52)	3,120 (52.5) 64 (1.08) 267 (4.49) 2,492 (41.93)	<.001
	Surgical Margin Status	No Yes Unknown	7,359 (97.2) 200 (2.64) 12 (0.16)	5,735 (96.5) 190 (3.2) 18 (0.3)	0.032
	Charlson-Deyo score	0 1 2+	4,969 (65.63) 1,794 (23.7) 808 (10.67)	4210 (70.84) 1,248 (21) 485 (8.16)	<.001
	Chemotherapy	No Yes Unknown	6,031 (79.66) 1,301 (17.18) 239 (3.16)	4,313 (72.57) 1,468 (24.7) 162 (2.73)	<.001
	Type of Surgery	Partial colectomy Subtotal colectomy/hemicolectomy Surgery NOS Total Colectomy	994 (13.13) 6394 (84.45) 4 (0.05) 179 (2.36)	3,588 (60.37) 2,065 (34.75) 13 (0.22) 277 (4.66)	<.001
	Age at Diagnosis	Mean Median Min Max Std Dev	68.41 69 21 90 13.23	63.69 64 18 90 13.64	<.001
	Tumor Size (cm)	Mean Median Min Max Std Dev	5.4 5 0.1 98.9 4.04	5.23 4.8 0.1 90 3.44	0.014
	**Covariate**	**Level**	**Right N = 2397**	**Left N = 774**	**P-value**
Stage III MSI-H Patients	Sex	Male Female	971 (40.51) 1,426 (59.49)	418 (54.01) 356 (45.99)	<.001
	Race	African American Other Caucasian	252 (10.51) 104 (4.34) 2,041 (85.15)	118 (15.25) 65 (8.4) 591 (76.36)	<.001
	Insurance Status	Government Unknown Not insured Private	1,544 (64.41) 24 (1) 102 (4.26) 727 (30.33)	367 (47.42) 11 (1.42) 41 (5.3) 355 (45.87)	<.001
	Surgical Margin Status	No Yes Unknown	2,157 (89.99) 229 (9.55) 11 (0.46)	697 (90.05) 65 (8.4) 12 (1.55)	0.005
	Charlson-Deyo score	0 1 2+	1,641 (68.46) 513 (21.4) 243 (10.14)	579 (74.81) 144 (18.6) 51 (6.59)	0.001
	Chemotherapy	No Yes Unknown	734 (30.62) 1,613 (67.29) 50 (2.09)	171 (22.09) 589 (76.1) 14 (1.81)	<.001
	Type of Surgery	Partial colectomy Subtotal colectomy/hemicolectomy Surgery NOS Total Colectomy	255 (10.64) 2061 (85.98) 1 (0.04) 80 (3.34)	435 (56.2) 290 (37.47) 1 (0.13) 48 (6.2)	<.001
	Age at Diagnosis	Mean Median Min Max Std Dev	67.59 70 18 90 15.49	60.55 61 18 90 15.65	<.001
	Tumor Size (cm)	Mean Median Min Max Std Dev	6.27 6 0.1 98.8 4.01	5.26 4.6 0.1 58 3.21	<.001
	**Covariate**	**Level**	**Right N = 7366**	**Left N = 6905**	**P-value**
Stage III MSS Patients	Sex	Male Female	3,487 (47.34) 3,879 (52.66)	3,584 (51.9) 3,321 (48.1)	<.001
	Race	African American Other Caucasian	1,086 (14.74) 404 (5.48) 5,876 (79.77)	882 (12.77) 545 (7.89) 5,478 (79.33)	<.001
	Insurance Status	Government Unknown Not insured Private	4,321 (58.66) 79 (1.07) 262 (3.56) 2,704 (36.71)	2,942 (42.61) 76 (1.1) 286 (4.14) 3,601 (52.15)	<.001
	Surgical Margin Status	No Yes Unknown	6,795 (92.25) 541 (7.34) 30 (0.41)	6,394 (92.6) 475 (6.88) 36 (0.52)	0.344
	Charlson-Deyo score	0 1 2+	5,020 (68.15) 1,620 (21.99) 726 (9.86)	5,145 (74.51) 1,271 (18.41) 489 (7.08)	<.001
	Chemotherapy	No Yes Unknown	1,848 (25.09) 5,366 (72.85) 152 (2.06)	1,265 (18.32) 5537 (80.19) 103 (1.49)	<.001
	Type of Surgery	Partial colectomy Subtotal colectomy/hemicolectomy Surgery NOS Total Colectomy	963 (13.07) 6,241 (84.73) 1 (0.01) 161 (2.19)	4,341 (62.87) 2,236 (32.38) 14 (0.2) 314 (4.55)	<.001
	Age at Diagnosis	Mean Median Min Max Std Dev	66.34 67 21 90 13.58	59.22 59 19 90 14.02	<.001
	Tumor Size (cm)	Mean Median Min Max Std Dev	5.21 4.8 0.1 98.9 4.19	4.49 4.2 0 98.9 2.45	<.001

### Tumor Side and Overall Survival

**Figure 2:**
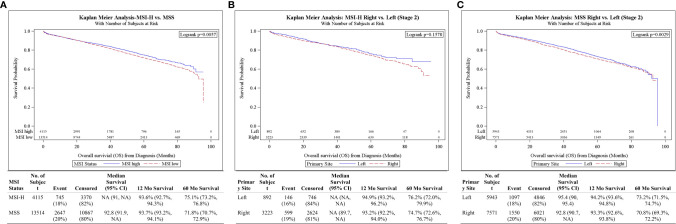
**(A)** Overall survival for Stage II patients by MSI status. **(B)** Overall survival for Stage II MSI-H patients by tumor side. **(C)** Overall survival for Stage II MSS patients by tumor side.

Stage II MSI-H had better 5-year OS compared to their MSS counterparts (75.1 *vs* 71.8%, p = 0.0057) (**Figure 2A**). On multivariable analysis, stage II MSI-H tumors were also associated with improved OS compared to MSS (HR 0.84, 95% CI 0.77–0.91, p < 0.001) (**Table 3**). There was no significant difference in survival between stage II MSI-H L *vs* R (5-year OS: 76.2 *vs* 74.7%, p = 0.1578) (**Figure 2B**). Stage II MSS CC 5-year OS was better in L *vs* R (73.2 *vs* 70.8%, p = 0.0029) (**Figure 2C**).

**Table 3:** Multivariate association with overall survival (Stages II and III).

Covariate	Level	Stage II Hazard Ratio (95% CI) p-value	Stage III Hazard Ratio (95% CI) p-value
Sex	Female Male*	0.75 (0.70–0.81) p < 0.001	0.82 (0.77–0.86) p < 0.001
Race	Caucasian Other/Unknown African American*	0.91 (0.81–1.02) p = 0.115 0.67 (0.54–0.83) p < 0.001	0.97 (0.89–1.07) p = 0.548 0.85 (0.72–0.99) p = 0.038
Primary Site	Left Right*	1.14 (1.05–1.24) p = 0.002	0.89 (0.83–0.96) p = 0.002
Microsatellite Instability (MSI) Status	MSI-H MSS*	0.84 (0.77–0.91) p < 0.001	0.96 (0.89–1.03) p = 0.259
Year of Diagnosis	2013–2015 2010–2012*	1.10 (1.02–1.19) p = 0.011	1.06 (0.99–1.12) p = 0.077
Insurance Status	Private Insurance Not Insured Unknown Government Insurance*	0.78 (0.71–0.87) p < 0.001 1.33 (1.06–1.67) p = 0.015 1.00 (0.68–1.47) p = 0.989	0.85 (0.79–0.92) p < 0.001 0.95 (0.80–1.12) p = 0.532 0.88 (0.66–1.17) p = 0.373
Charlson**–**Deyo Score	2+ 1 0*	1.92 (1.75–2.11) p < 0.001 1.29 (1.20–1.40) p < 0.001	1.64 (1.51–1.78) p < 0.001 1.23 (1.15–1.32) p < 0.001
Chemotherapy	Unknown Yes No*	0.73 (0.58–0.91) p = 0.006 0.68 (0.60–0.76) p < 0.001	0.55 (0.45–0.68) p < 0.001 0.33 (0.31–0.35) p < 0.001
Surgery at Primary Site	Total colectomy Surgery NOS Subtotal colectomy/hemicolectomy Partial colectomy*	1.38 (1.15–1.66) p < 0.001 1.15 (0.48–2.79) p = 0.752 1.07 (0.98–1.16) p = 0.158	1.46 (1.26–1.69) p < 0.001 1.38 (0.62–3.09) p = 0.434 1.04 (0.96–1.12) p = 0.318
Surgical Margin Status	Unknown Yes No*	1.15 (0.62–2.15) p = 0.662 1.87 (1.60–2.18) p < 0.001	2.50 (1.78–3.51) p < 0.001 1.82 (1.67–1.98) p < 0.001
Age at Diagnosis		1.05 (1.05–1.06) p < 0.001	1.02 (1.02–1.02) p < 0.001

*Reference.

**Figure 3:**
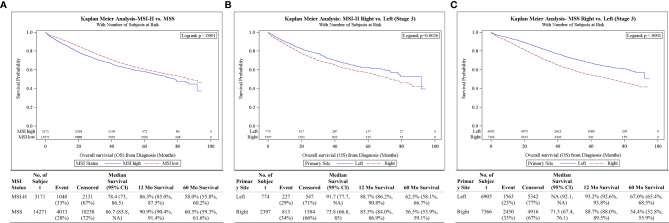
**(A)** Overall survival for Stage III patients by MSI status. **(B)** Overall survival for Stage III MSI-H patients by tumor side. **(C)** Overall survival for Stage III MSS patients by tumor side.

For stage III CC, survival was better in MSS compared to MSI-H (5-year OS: 60.5 *vs* 58.0%, p < 0.001) (**Figure 3A**). However, after the adjustments of potential confounders in multivariable analysis, stage III MSI-H tumors were no longer associated with OS difference compared to MSS (HR 0.96, 95% CI 0.89–1.03, p = 0.259) (**Table 3**). Stage III MSI-H CC survival was better in L *vs* R (5-year OS 62.5 *vs* 56.5%, p = 0.0026) (**Figure 3B**). Stage III MSS CC survival was better in L *vs* R (5-year OS 67.0 vs 54.4%, p < 0.001) (**Figure 3C**).

### Adjuvant Chemotheraand Overall Survival

For stage II MSI-H patients, 22.2% (n = 198/892) of left-sided and 12.5% (n = 404/3,223) of right-sided patients received AC. For stage II MSS patients, 24.7% (n = 1,468/5,943) of the left-sided and 17.2% (n =1,301/7,571) of the right-sided patients received AC (**Table 2**). Survival benefit from AC was observed for stage II right-sided MSI-H patients (5-year OS 83.6 *versus* 73.3%; p = 0.0013) (**Figure Supp 1A**), left-sided MSS patients (5-year OS 84.6 *versus* 69.3%; p < 0.0001) (**Figure Supp 1B**) and right-sided MSS patients (5-year OS 82.9 *versus* 67.9%; p < 0.0001) (**Figure Supp 1C**). No survival benefit from AC was observed for stage II left-sided MSI-H patients (5-year OS 76.1 *versus* 76.3%; p = 0.3147) (**Figure Supp 1D**). Multivariate analysis with adjustment of potential confounders demonstrated the same findings (data not presented).

For stage III MSI-H patients, 76.1% (n = 589/774) of left-sided and 67.3% (n =1,613/2,397) of right-sided patients received AC. For stage III MSS patients, 80.2% (n = 5,537/6,905) of left-sided and 73.0% (n = 5,376/7,366) of right-sided patients received AC (**Table 2**). Survival benefit from AC was observed for stage III left-sided MSI-H patients (5-year OS 70.5 *versus* 30.7%, p < 0.0001) (**Figure Supp 2A**), right-sided MSI-H patients (5-year OS 65.2 *versus* 37.5%; p < 0.0001) (**Figure Supp 2B**), left-sided MSS patients (5-year OS 74.7 *versus* 33.1%; p < 0.0001) (**Figure Supp 2C**) and right-sided MSS patients (5-year OS 63.6 *versus* 28.1%; p < 0.0001) (**Figure Supp 2D**). Multivariate analysis with adjustment of potential confounders demonstrated the same findings (data not presented).

## Discussion

The results of this study demonstrate that survival was better in the left compared to right-sided tumors among stage II MSS, stage III MSS, and stage III MSI-H CC patients. In stage II MSI-H CC, there was no difference in survival among the left *versus* right-sided tumors. This study confirms and emphasizes previous reports that bearing a left-sided tumor was associated with significantly improved survival (2, 5, 6). In two different SEER-Medicare studies, right-sided stage II cancers had higher overall survival than left-sided cancers and right-sided stage III CC had lower overall survival than left-sided CC (4, 6). However, similar to prior studies, they did not have MSI status of the tumors. Results from a recent meta-analysis of 66 studies concluded that tumors originating in the left side of the colon were significantly associated with an absolute 19% reduced risk of death (2). Such a survival benefit was independent of race, stage (II, III, IV), year of publication, and type of study (2). Several studies have found that patients with MSI-H tumors have an improved prognosis and that MSI status is an independent predictor of overall survival (19–23). MSI-H is mostly seen in right-sided CC (17), with less than 5% seen in left-sided CC (14). The stage profile of MSI-H tumors is also more favorable (4). It is estimated that MSI-H accounts for 20–25% of stage II right-sided cancers and 15% of stage III right-sided tumors (28). MSI-H tumors have also been associated with a decreased risk of lymph node and distant organ metastases; providing further evidence that right-sided stage III cancers may be more biologically distinct from right-sided stage II cancers (22). Thus, primary tumor location can be used as a prognostic tool in CC in clinical decision-making processes especially with known MSI status as described in this study.

The results of this study demonstrated that there was no survival benefit from AC for stage II left-sided MSI-H patients; however, survival benefit from AC was observed for stage II right-sided MSI-H patients, left- and right-sided stage II MSS patients. Significantly more patients with left sided tumors received chemotherapy in all groups and the same survival findings were seen after adjustment of potential confounders by multivariate analysis. These results differed from those reached by Weiss et al., whereby no survival benefit was seen for either stage II right-sided or left-sided CC patients who received AC compared to those who did not (29). Instead of MSI status, Weiss et al. utilized right-sided tumor location as a surrogate for MSI status and included only Medicare patients age 66 and older. The current study differs significantly as it has MSI status of all the patients age 18 and older included in the analysis. Interestingly in this study, left-sided stage II patients received AC more often, similarly demonstrated in the study by Weiss et al. Consistent with previous reports, this study shows a significant survival benefit for stage III patients who receive AC, regardless of tumor location and MSI status (29–38). Survival benefit from AC is established for stage III CC (13); however, uncertainty exists for stage II patients (29). In resected stage II CC, the presence of MSI has been associated with a more favorable prognosis and lack of benefit from fluorouracil-based AC (39). Sinicrope et al. evaluated the prognostic impact of MSI status in patients with stage III CC enrolled in a randomized trial of FOLFOX-based AC and found that MSI-H proximal tumors (right-sided) had favorable disease free survival compared to MSS (40). In their analysis of five previous randomized trials of fluorouracil based AC, Ribic et al. found that there was no benefit from AC in stage II and III MSI-H CC in contrast to a benefit seen in MSS tumors (19). Given the previously identified relationship between tumor location and clinical outcomes without known MSI status, we sought to determine the impact of tumor location with known MSI status on the clinical outcomes of stage II and III CC patients.

To the best of our knowledge, this is the first study that describes the site of CC (right *vs* left) as an independent prognostic factor in the presence of known MSI status in stage II and III CC. This eliminates the potential bias associated with conclusions reached by other studies that utilized tumor location as a surrogate for MSI status. Despite the uniqueness of the analysis, this is a retrospective study with its inherent limitations. Patient treatment preferences and physician practice patterns are unmeasured factors that may play a role in clinical outcomes. Results of this study could be subject to unmeasured confounding particularly if physician practice patterns are influenced by tumor location. The limitations of this study also include lack of specific chemotherapy regimen data, duration of chemotherapy, and data about adverse effects of chemotherapy. The analysis was primarily based on receipt of any chemotherapy and does not account for early discontinuation of prescribed treatment, which possibly could impact the survival benefit. In addition, disease-specific mortality, recurrence indices, and response to treatment are not captured in the NCDB. Despite these limitations, this study demonstrated the independent prognostic significance of CC side in the presence of known MSI status. Based on the results of this study, the side of origin of CC (left *vs* right) should be acknowledged as a criterion for establishing prognosis in stage II and III disease and could impact decisions regarding treatment of patients with CC. Moreover, the results of this study can assist providers in the treatment decision for stage II CC patients in which routine AC is not established, and primary tumor location might represent an important stratification factor for future adjuvant clinical trials.

## Conclusion

This large national cancer database analysis revealed that survival was better in left-sided tumors compared to right in stage II MSS, stage III MSS, and stage III MSI-H CC. Survival benefit from adjuvant chemotherapy was observed in all patients except in stage II left-sided MSI-H CC patients.

## Data Availability Statement

The original contributions presented in the study are included in the article/**Supplementary Material**. Further inquiries can be directed to the corresponding author.

## Ethics Statement

No ethical approval was required for the study as de-identified patient information in the NCDB is legally accessible to the public.

## Author Contributions

MA conceptualized the study, conducted the data curation, performed the formal analysis, conducted the investigation, was in charge of the project administration, supervised the study, conducted the validation, and wrote, reviewed, and edited the article. KZ conceptualized the study, conducted the data curation, performed the formal analysis, conducted the investigation, was in charge of the project administration, supervised the study, conducted the validation, and wrote, reviewed, and edited the article. RJ conducted the data curation, developed the methodology, provided the software, wrote the original draft, and wrote, reviewed, and edited the article. SW conducted the data curation, developed the methodology, provided the software, wrote the original draft, and wrote, reviewed, and edited the article. OA developed the methodology, conducted the investigation, wrote the original draft, and wrote, reviewed, and edited the article. WS developed the methodology, conducted the investigation, wrote the original draft, and wrote, reviewed, and edited the article. CW developed the methodology, conducted the investigation, wrote the original draft, and wrote, reviewed, and edited the article. MB conceptualized the study, was in charge of the project administration, conducted the investigation, validated the study, and reviewed and edited the article. BE-R developed the methodology, conducted the investigation, wrote the original draft, and wrote, reviewed, and edited the article. All authors contributed to the article and approved the submitted version.

## Conflict of Interest

The authors declare that the research was conducted in the absence of any commercial or financial relationships that could be construed as a potential conflict of interest.

## Supplementary Material

Supplementary Figure 1 **(A)** Overall Survival for Stage II MSI-H Right-Sided Tumors by Receipt of AC. **(B)** Overall Survival for Stage II MSS Left-Sided Tumors by Receipt of AC. **(C)** Overall Survival for Stage II MSS Right-Sided Tumors by Receipt of AC. **(D)** Overall Survival for Stage II MSI-H Left-Sided Tumors by Receipt of AC.

Supplementary Figure 2 **(A)** Overall Survival for Stage III MSI-H Left-Sided Tumors by Receipt of AC. **(B)** Overall Survival for Stage III MSI-H Right-Sided Tumors by Receipt of AC. **(C)** Overall Survival for Stage III MSS Left-Sided Tumors by Receipt of AC. **(D)** Overall Survival for Stage III MSS Right-Sided Tumors by Receipt of AC.
