# Supplementary material for: Impact of Tumor Side on Clinical Outcomes in Stage II and III Colon Cancer With Known Microsatellite Instability Status
Source: Front Oncol. 2021 Mar 30;11:592351. doi: 10.3389/fonc.2021.592351 (PMC8042136; doi:10.3389/fonc.2021.592351)
Supplement: Supplementary Figure 1 — (A) Overall Survival for Stage II MSI-H Right-Sided Tumors by Receipt of AC. (B) Overall Survival for Stage II MSS Left-Sided Tumors by Receipt of AC. (C) Overall Survival for Stage II MSS Right-Sided Tumors by Receipt of AC. (D) Overall Survival for Stage II MSI-H Left-Sided Tumors by Receipt of AC. [file DataSheet_1.pdf]

Figure Supplement 1A:

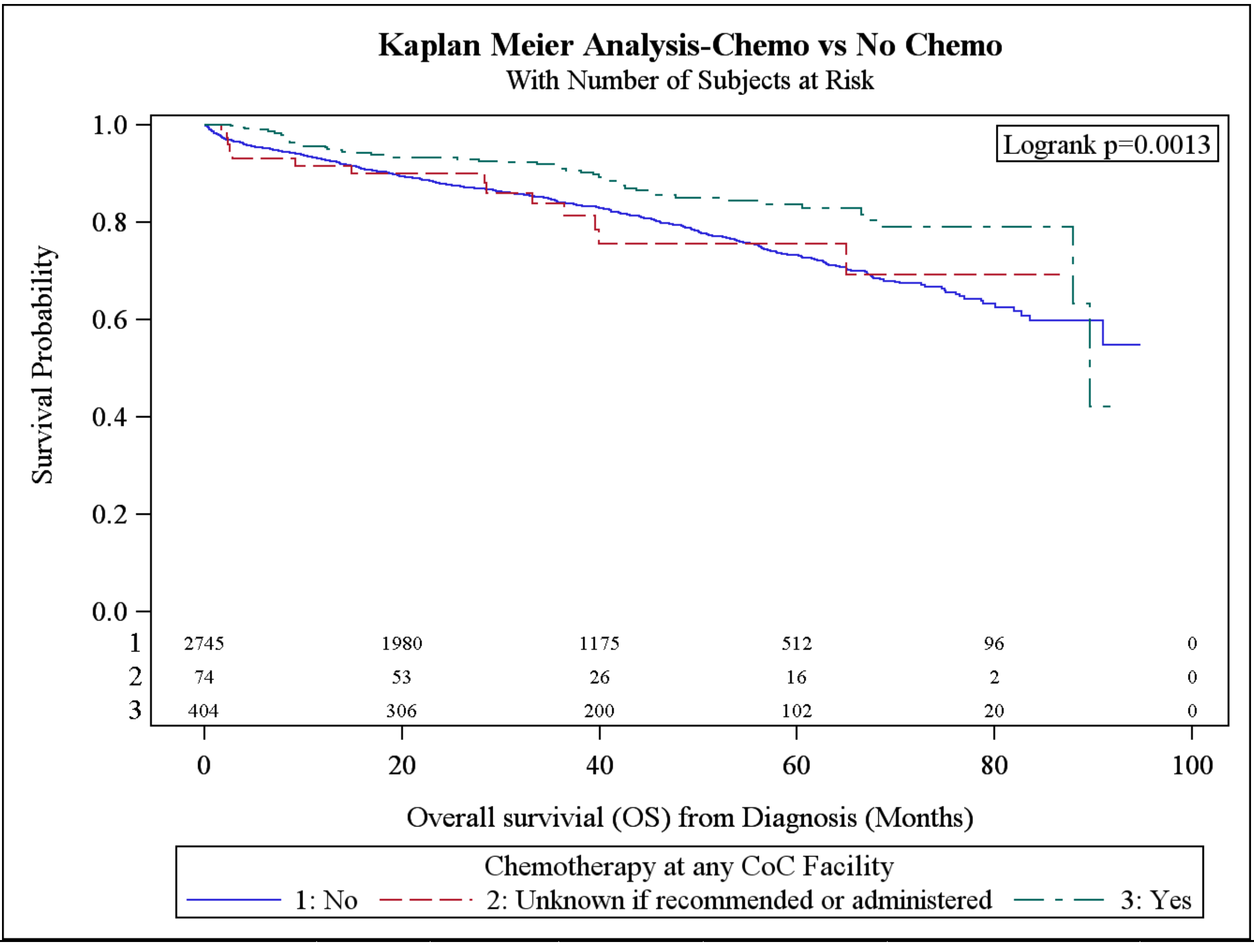

Chemotherapy at any CoC Facility	No. of Subject	Event	Censored	Median Survival (95% CI)	12 Mo Survival	60 Mo Survival
No	2745	533 (19%)	2212 (81%)	NA (91, NA)	92.9% (91.8%, 93.8%)	73.3% (70.9%, 75.5%)
Unknown if recommended or administered	74	14 (19%)	60 (81%)	NA (65, NA)	91.6% (82.2%, 96.1%)	75.5% (60.5%, 85.5%)
Yes	404	52 (13%)	352 (87%)	89.7 (87.9, NA)	95.5% (92.9%, 97.2%)	83.6% (78.4%, 87.7%)

Figure Supplement 1B:

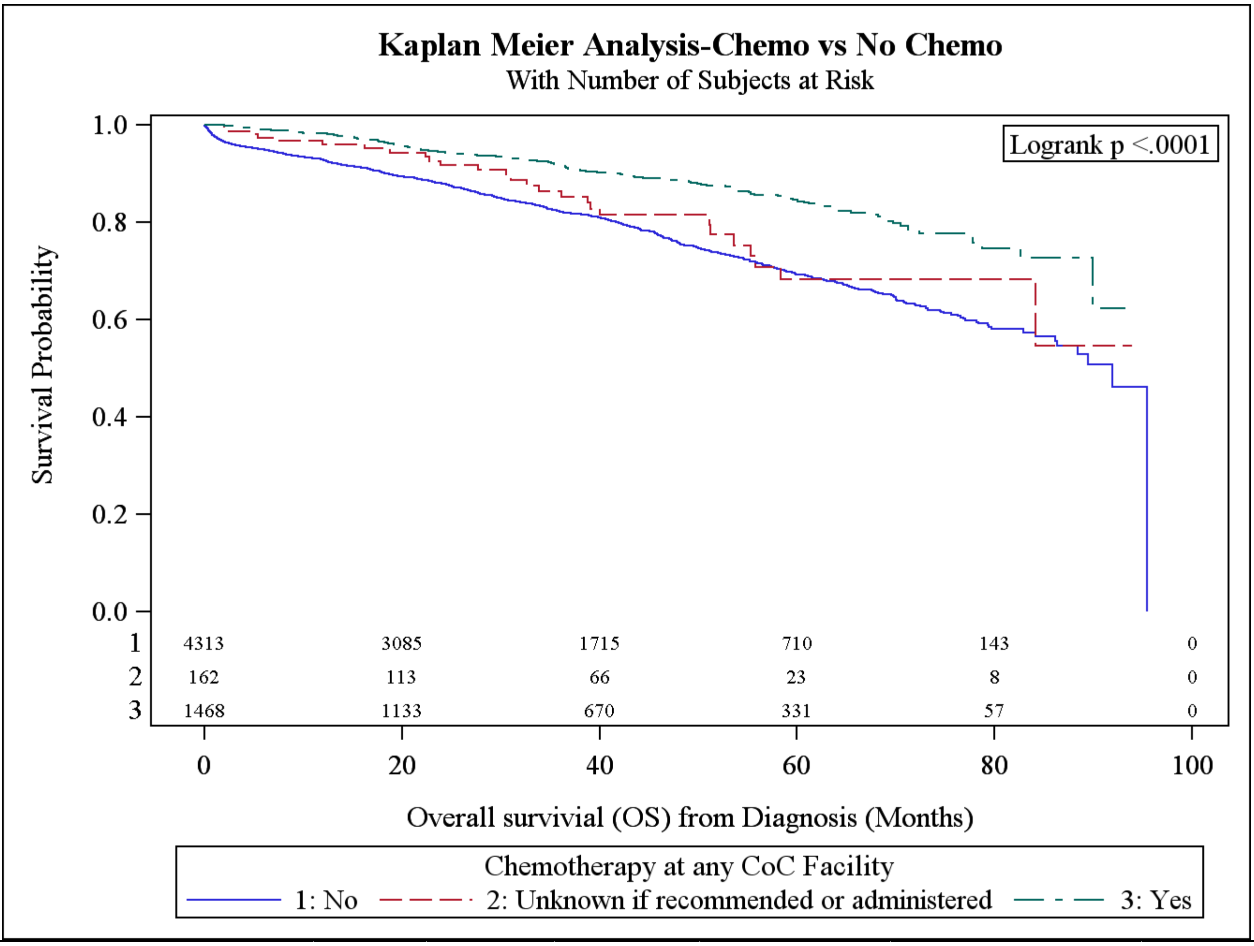

**Chemotherapy at any CoC Facility**	**No. of Subject**	**Event**	**Censored**	**Median Survival (95% CI)**	**12 Mo Survival**	**60 Mo Survival**
No	4313	908 (21%)	3405 (79%)	91.9 (86.4, 95.4)	92.8% (92.0%, 93.5%)	69.3% (67.2%, 71.2%)
Unknown if recommended or administered	162	27 (17%)	135 (83%)	NA (84.1, NA)	95.9% (91.1%, 98.1%)	68.3% (55.1%, 78.3%)
Yes	1468	162 (11%)	1306 (89%)	NA (90, NA)	98.2% (97.4%, 98.8%)	84.6% (81.8%, 87.0%)

Figure Supplement 1C:

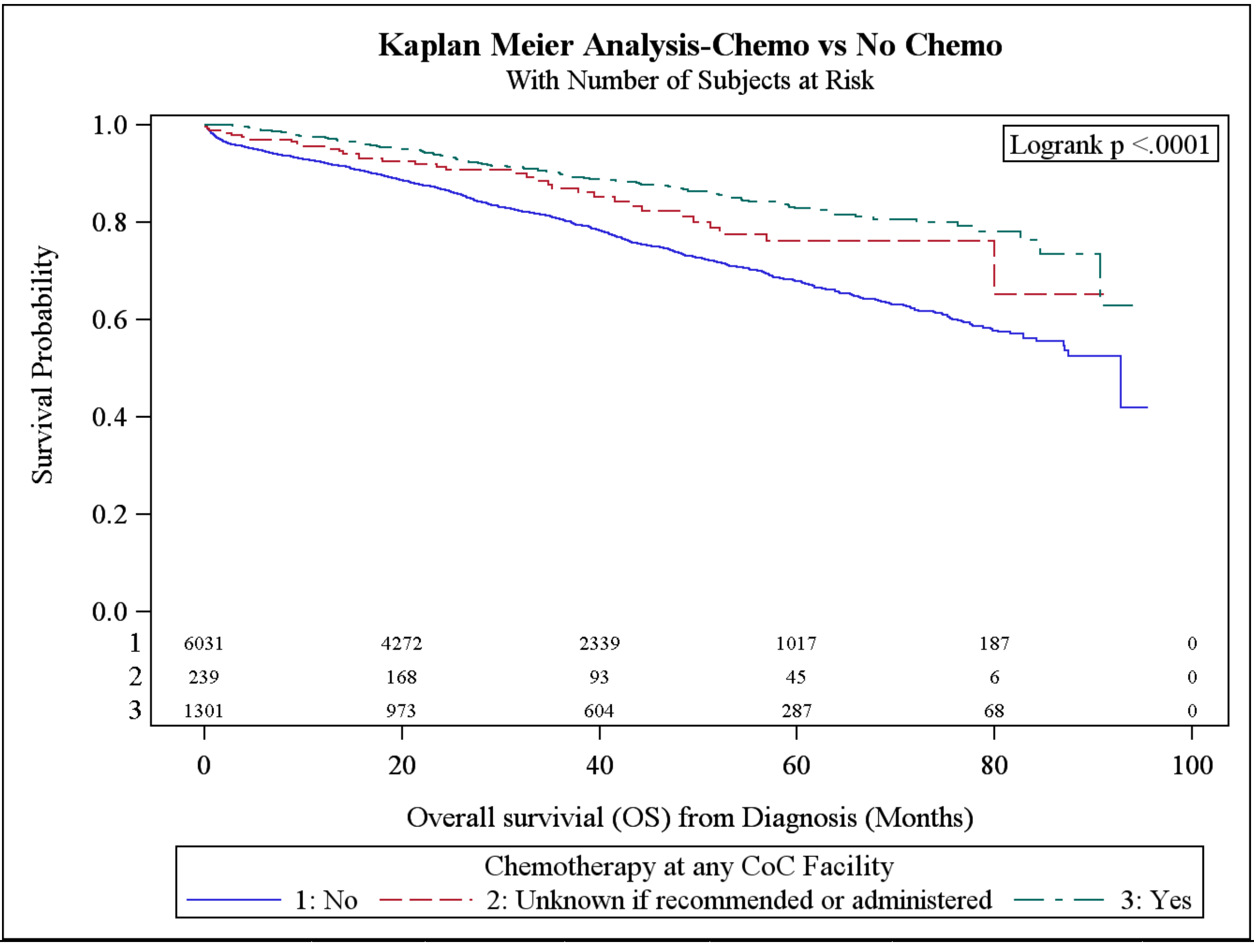

Chemotherapy at any CoC Facility	No. of Subject	Event	Censored	Median Survival (95% CI)	12 Mo Survival	60 Mo Survival
No	6031	1362 (23%)	4669 (77%)	92.8 (87.1, NA)	92.3% (91.5%, 92.9%)	67.9% (66.2%, 69.5%)
Unknown if recommended or administered	239	35 (15%)	204 (85%)	NA (80, NA)	95.5% (91.8%, 97.6%)	76.1% (67.1%, 82.9%)
Yes	1301	153 (12%)	1148 (88%)	NA (90.7, NA)	97.4% (96.4%, 98.2%)	82.9% (79.8%, 85.5%)

Figure Supplement 1D:

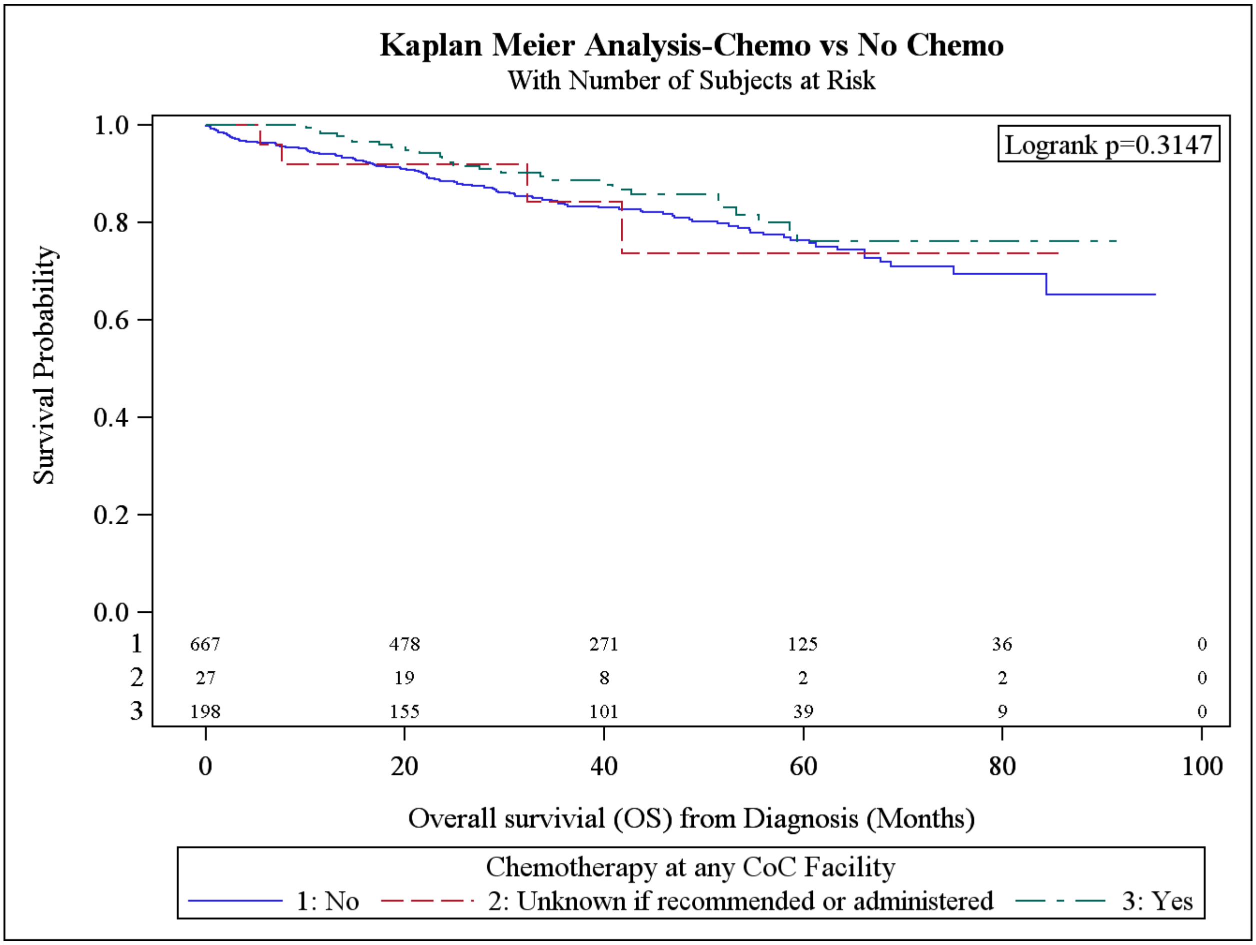

Chemotherapy at any CoC Facility	No. of Subject	Event	Censored	Median Survival (95% CI)	12 Mo Survival	60 Mo S[illegible]
No	667	115 (17%)	552 (83%)	NA (NA, NA)	94.0% (91.8%, 95.6%)	76.3% [illegible] 80.[illegible]
Unknown if recommended or administered	27	4 (15%)	23 (85%)	NA (41.8, NA)	91.8% (71.1%, 97.9%)	73.7% [illegible] 90.[illegible]
Yes	198	27 (14%)	171 (86%)	NA (NA, NA)	98.3% (94.9%, 99.5%)	76.1% [illegible] 83.[illegible]

Figure Supplement 2A:

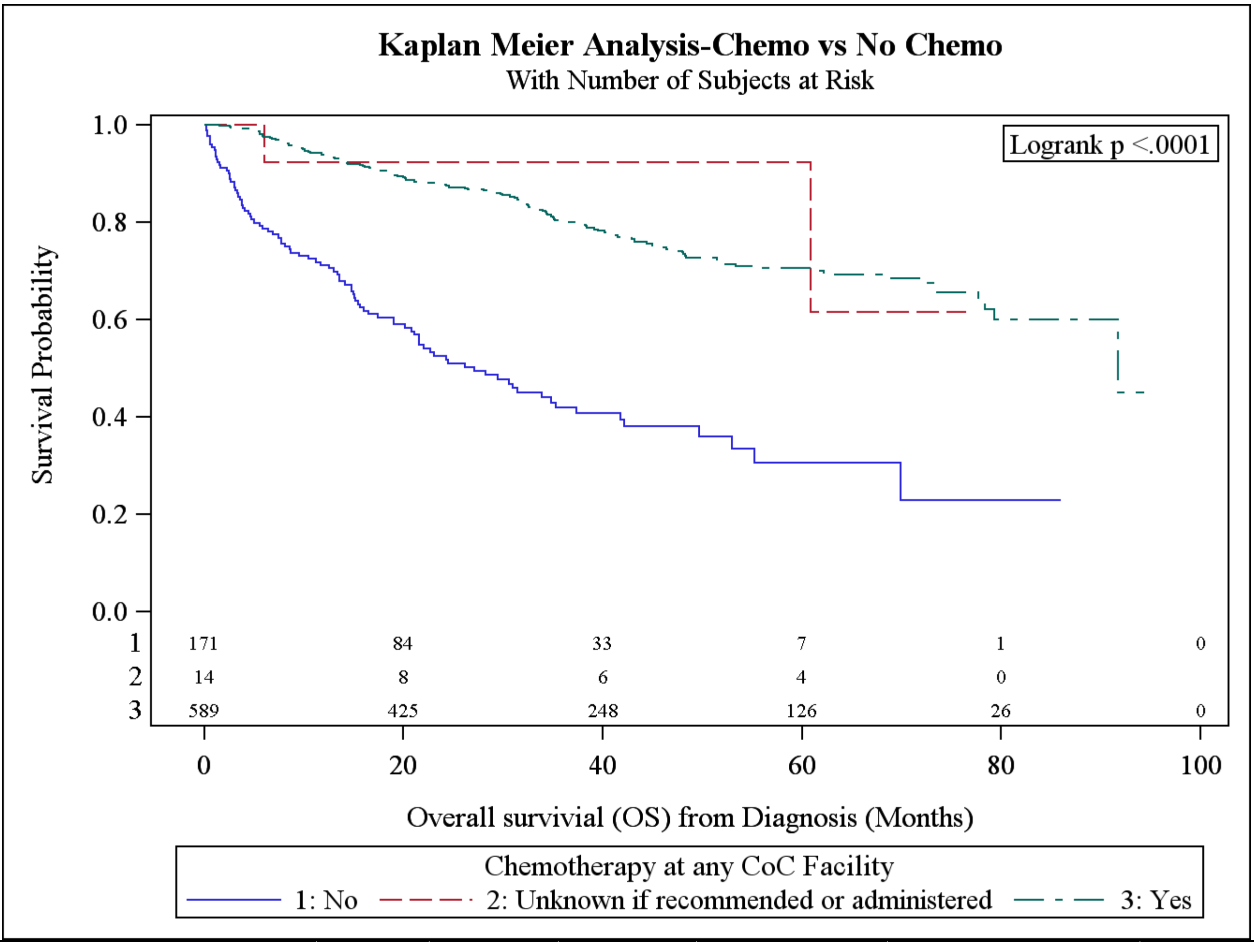

Chemotherapy at any CoC Facility	No. of Subject	Event	Censored	Median Survival (95% CI)	12 Mo Survival	60 Mo Survival
No	171	94 (55%)	77 (45%)	27.1 (20.8, 37.4)	71.2% (63.6%, 77.4%)	30.7% (20.7%, 41.1%)
Unknown if recommended or administered	14	2 (14%)	12 (86%)	NA (60.9, NA)	92.3% (56.6%, 98.9%)	92.3% (56.6%, 98.9%)
Yes	589	131 (22%)	458 (78%)	91.7 (91.7, NA)	93.7% (91.4%, 95.5%)	70.5% (65.5%, 75.0%)

Figure Supplement 2B:

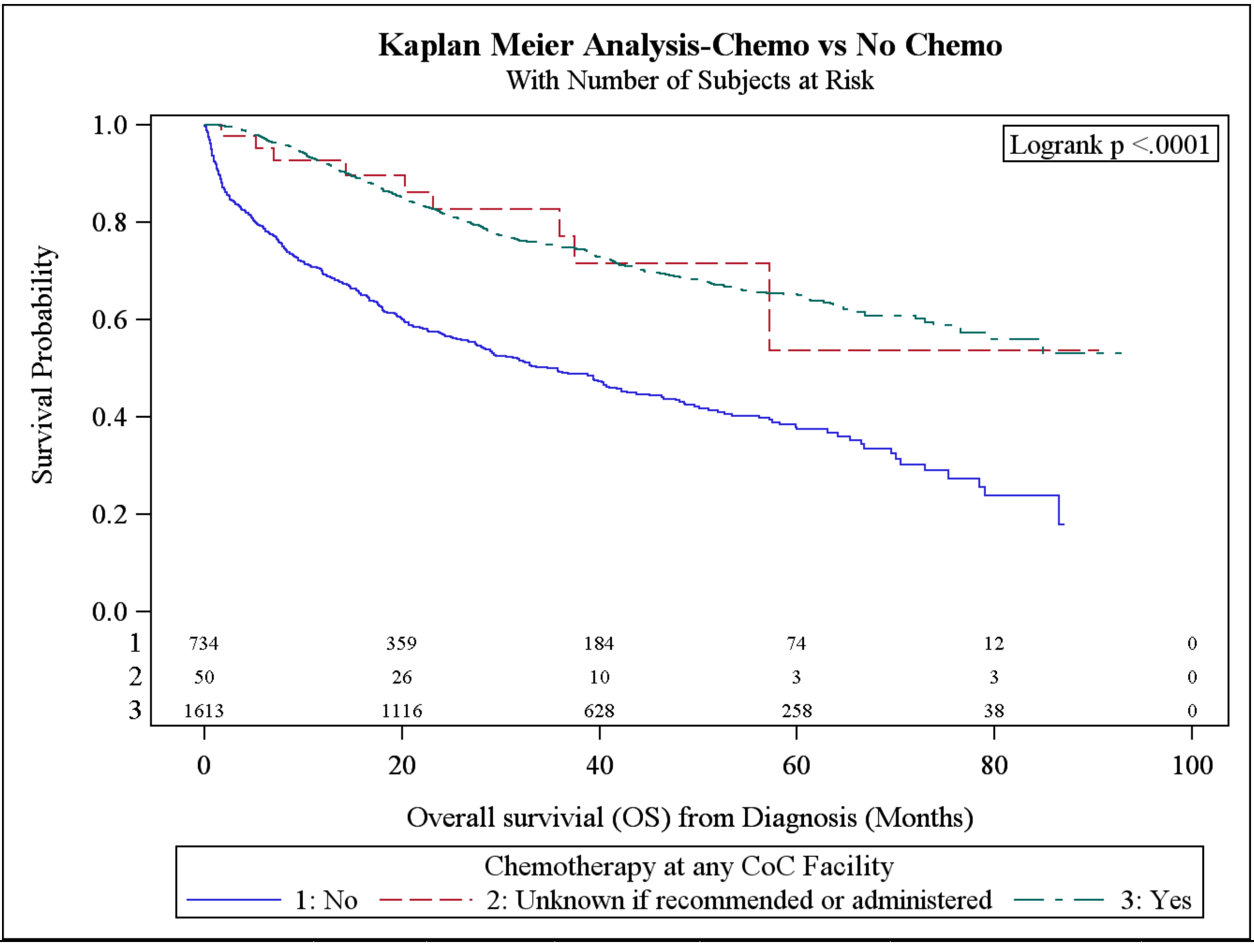

Chemotherapy at any CoC Facility	No. of Subject	Event	Censored	Median Survival (95% CI)	12 Mo Survival	60 Mo Survival
No	734	380 (52%)	354 (48%)	34.7 (28.5, 41.8)	69.5% (65.9%, 72.7%)	37.5% (32.8%, 42.1%)
Unknown if recommended or administered	50	9 (18%)	41 (82%)	NA (37.5, NA)	92.6% (78.9%, 97.6%)	53.7% (18.6%, 79.5%)
Yes	1613	424 (26%)	1189 (74%)	NA (85, NA)	92.6% (91.1%, 93.8%)	65.2% (62.1%, 68.1%)

Figure Supplement 2C:

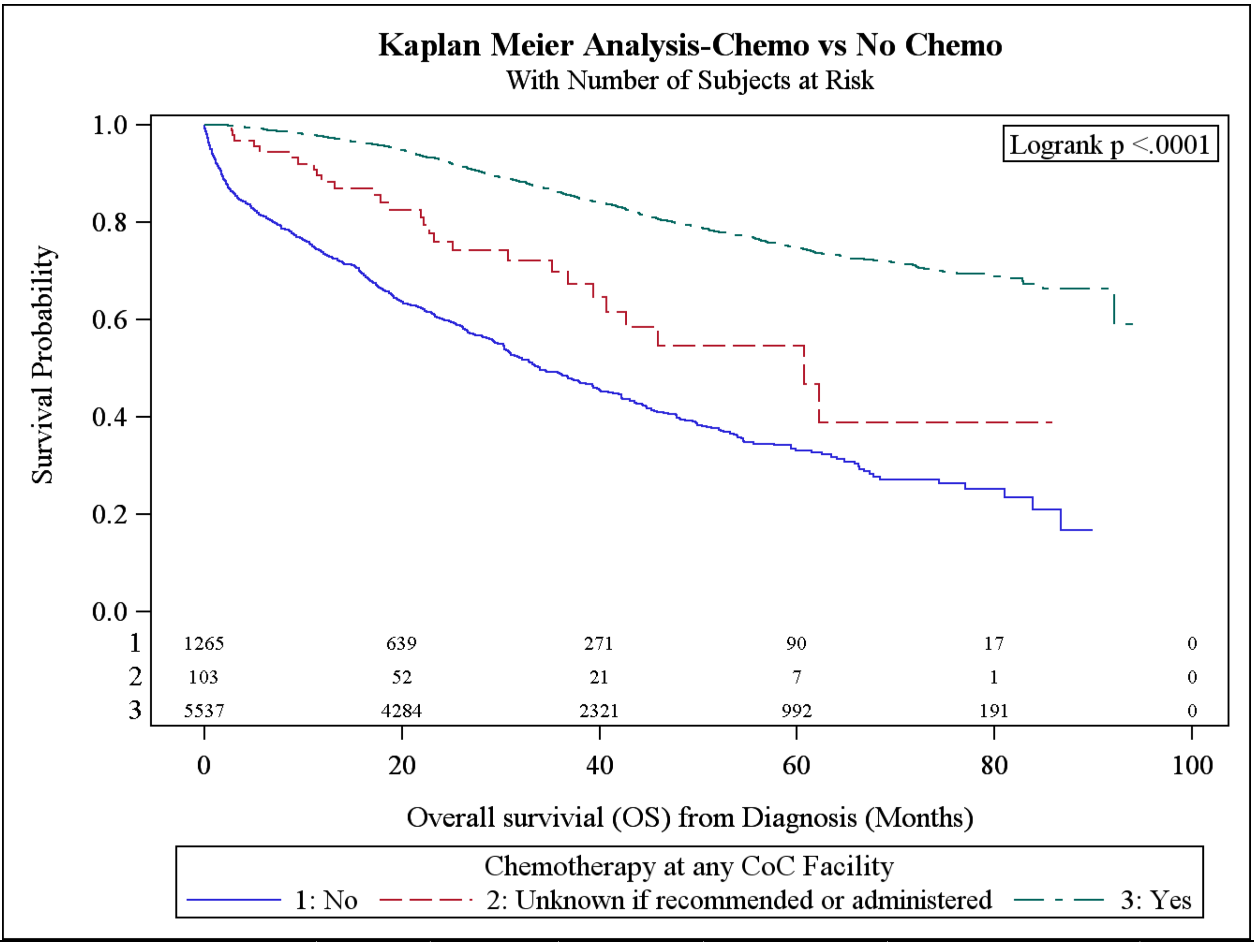

Chemotherapy at any CoC Facility	No. of Subject	Event	Censored	Median Survival (95% CI)	12 Mo Survival	60 Mo Survival
No	1265	641 (51%)	624 (49%)	33.9 (31, 38.6)	73.8% (71.2%, 76.2%)	33.1% (29.4%, 36.9%)
Unknown if recommended or administered	103	28 (27%)	75 (73%)	60.7 (40.7, NA)	88.3% (79.3%, 93.5%)	54.6% (38.5%, 68.0%)
Yes	5537	894 (16%)	4643 (84%)	NA (92.1, NA)	97.6% (97.2%, 98.0%)	74.7% (73.0%, 76.3%)

Figure Supplement 2D:

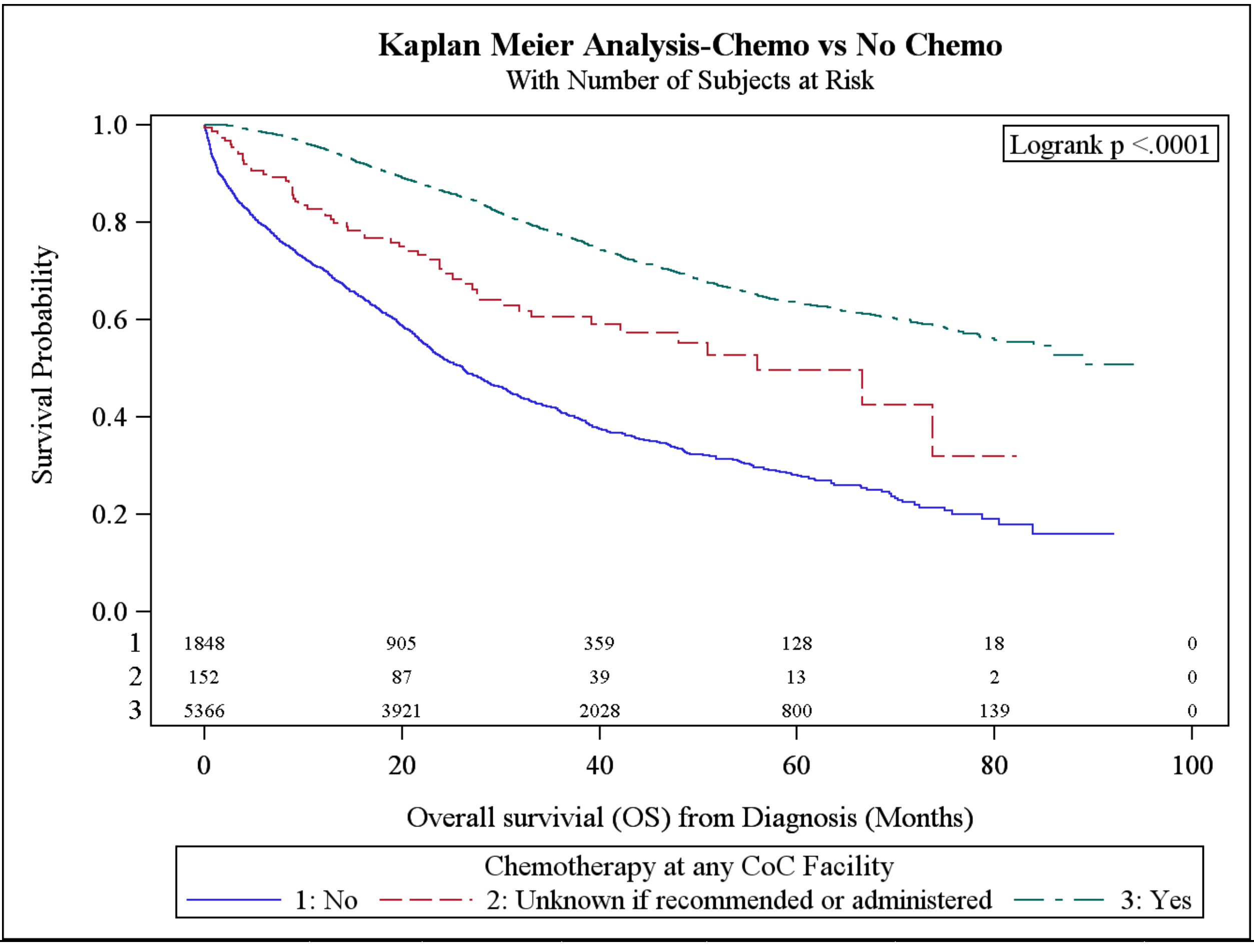

Chemotherapy at any CoC Facility	No. of Subject	Event	Censored	Median Survival (95% CI)	12 Mo Survival	60 Mo Survival
No	1848	1076 (58%)	772 (42%)	26.2 (23.9, 28.3)	70.3% (68.1%, 72.4%)	28.1% (25.3%, 30.9%)
Unknown if recommended or administered	152	56 (37%)	96 (63%)	56 (39.2, NA)	82.0% (74.7%, 87.4%)	49.7% (37.6%, 60.7%)
Yes	5366	1318 (25%)	4048 (75%)	NA (85.8, NA)	95.2% (94.6%, 95.7%)	63.6% (61.8%, 65.4%)
